# Mr. Mayo's Case of Ruptured Uterus

**Published:** 1813-12

**Authors:** Charles Mayo

**Affiliations:** Winchester


*To the Editor of the Medical and Physical Journal.*

SIR,

SEEING a case of "Rupture of the Uterus during Labour" in your Journal for this month, by Dr. Ramsbotham, I was reminded of one which occurred to me not long since.

I have the honor to be

*Winchester,* Your obedient Servant,

*Oct.* 14*th,* 1813. CHARLES MAYO.

Thursday, 17th of June last, I was called to Rachel Marsh, at five in the morning, and found that her water had broke, about an hour before, which waked her from a sound sleep. This, however, was succeeded by no pain, and therefore, after remaining with her a short time, I left her, desiring to be sent for when her pains commenced. I saw her again in the course of the day, but found that no pain of consequence had occurred, the water still continuing to come away. On Saturday I called again; she continued much the same, had had occasional pains, but passed her nights pretty quietly. On Monday morning I again saw her: the pains had been more frequent and severe, much water had drained from her, and early this morning a quantity of very fœtid and discolored discharge had come away. She felt great pain in the left inguinal region, and had experienced more or less in that situation throughout her pregnancy. In the evening, about nine, I was sent for in great haste, with intelligence that her labour-pains had now come on with great severity, and that the nurse expected

---

the the birth of the child very speedily. On my arrival, she was walking about, and several sharp pains came on soon after. I found the os uteri dilated to about the size of a crown piece, and very little below the brim of the pelvis, the head presenting. She was extremely restless, and would neither lay, stand, or sit, for a moment. After I had been with her a quarter of an hour, a sort of convulsive fit succeeded one of her pains; and I understood she had been subject to fits in her former labours. She was very short, and extremely corpulent, which, added to her pregnancy, rendered her very unwieldy, and, from her great restlessness, almost unmanageable. She continually exclaimed she should die, and begged that more assistance might be sent for. She had been attended by two other gentlemen in her former labours, and I requested that one of these might be fetched. In the mean time, as she sat, a pain came on, which was succeeded by a considerable rigor, and she became faint. Presently, on her rising from the seat, I perceived on the floor a quantity of blood. I immediately got her on the bed to examine: I found the os uteri much more dilated, but the head had made but little progress in its descent, and there was no hæmorrhage perceptible. As she laid down, she repeatedly said, I am going, and prayed for her children, as if she was sensible of her approaching end, which, from the frequency of these exclamations in the time of labour, no one had as yet regarded in a serious light; but now indeed both myself and her attendants began to be alarmed, when her extremities became cold, and she uttered a deep groan, and scarce five minutes had elapsed since she laid down, before she expired. The gentleman who had been sent for arrived soon after: he suggested a variety of accidents which might have been the causes of this event, and which we determined, if possible, to ascertain by dissection the succeeding day.

On dividing the parietes of the abdomen, a quantity of fluid tinged with blood escaped. The uterus occupied its natural situation, and at first sight appeared sound, but on raising the fundus, a large black echymosed patch was observed on the left and posterior part of the neck; here a limb of the child was felt to protrude, covered only by what appeared to be the peritoneal coat of the uterus, but so loaded with blood as not to enable us distinctly to ascertain. Without disturbing this, I laid open the anterior part of the uterus; the head of the child plugged up the superior aperture of the pelvis, and was descending in the natural way, and this we did not withdraw, in order to prevent the escape of the fluids with which we were now encumbered. We discovered discovered a distinct laceration in the inside of the uterus, at the place before mentioned, and the elbow and shoulder of the child were entangled in the opening. The supposed membranous covering easily gave way, and the naked arm was readily drawn through the fissure. With regard to the nature of this aperture, whether it was the effect of a momentary cause, or whether the result of long preceding disease, the opinions of several surgeons present differed. The edge of the opening, which was a sort of longitudinal slit about four or five inches in extent, appeared smooth and defined on one side; but being called away suddenly, neither time nor convenience would suffer me to make a more accurate investigation. No one doubted that this must have been the cause of her death. There had been no hæmorrhage of importance, but we were inclined to think the structure of the uterus somewhat depraved.

She had been the mother of ten children, which she had borne very quickly. I learnt from the gentlemen who had formerly attended her, and who were present at the examination, that she was always of that restless disposition during her labours. The early rupture of the membranes was probably in this case very prejudicial; and it has occurred to me to observe in several cases which have been related, that the subjects of them have been very corpulent, and have borne many children. From these causes, I conceive the muscular structure of the parietes of the abdomen frequently becomes very much debilitated, so as to diminish, and almost abolish its contractile power, and consequently deprive the uterus of a very powerful assistant during labour. The linea alba in this case was very wide, and the recti muscles pale and thin, spreading on each side to a great extent.

